# Optimizing the microbial synthesis of silver nanoparticles using *Gloeophyllum striatum* and their antimicrobial potential evaluation

**DOI:** 10.1038/s41598-023-48414-9

**Published:** 2023-11-30

**Authors:** Aleksandra Tończyk, Katarzyna Niedziałkowska, Katarzyna Lisowska

**Affiliations:** 1 Department of Industrial Microbiology and Biotechnology, Faculty of Biology and Environmental Protection, University of Lodz, 12/16 Banacha Street, 90-237 Lodz, Poland; 2 The BioMedChem Doctoral School of University of Lodz and Lodz Institutes of Polish Academy of Sciences, University of Lodz, 21/23 Matejki Street, 90-237 Lodz, Poland

**Keywords:** Biotechnology, Microbiology

## Abstract

The search for new sources of silver nanoparticles (AgNPs) is highly relevant in many fields. Mycosynthesis seems to be advantageous for large-scale production, and using brown rot fungi might be a promising solution. In this study, AgNP synthesis using *Gloeophyllum striatum* DSM 9592 was performed under various process conditions. The resulting AgNPs were characterized using UV/Vis, FT-IR, SEM and NTA techniques and their biological activities were determined. It was found that different synthesis conditions changed the production efficiency, which was the highest in 28 s AgNPs. Moreover, temperature and shaking conditions slightly affected the activity of the resulting AgNP types. Gram-negative bacteria were generally more susceptible to the action of AgNPs with MIC values two- or three-fold lower compared to Gram-positive strains. *Pseudomonas aeruginosa* was the most sensitive among tested strains with a MIC value of 1.56 µg/ml. The research was additionally extended by the biofilm formation assay for this strain. It was found that AgNPs of all types led to a reduction in biofilm-forming capability of *P. aeruginosa* over the tested concentration range. Haemolytic and cytotoxic activity assays showed that synthesis conditions also affected AgNP toxicity. For instance, 4 ns AgNPs were the least cytotoxic and cause less than 50% reduction of fibroblast viability in the concentration that inhibits the growth of *P. aeruginosa* completely. These results highlight the possible utility of mycogenic silver nanoparticles as an antibacterial agent in antiseptics or other external treatments.

## Introduction

In recent years, nanotechnology has received extensive attention as a rapidly developing, multidisciplinary field impacting the industry, agriculture, and pharmacology areas^1–3^. Nanomaterials can exhibit novel physical, chemical, and mechanical properties due to the ‘nano’ dimension. That property makes them more versatile than their initial bulk materials^4,5^. Thus, it is considered that nanomaterials as products of nanotechnology will determine the future of science^3^.

There are three strategies of metal nanoparticle (NP) synthesis, namely, physical, chemical, and biological. Physical and chemical routes are considered environmentally harmful, time-consuming, and expensive because of the necessity to use hazardous chemicals or provide special conditions^3,5,6^. Biological methods, also described as ‘green synthesis’, constitute the opposite characteristics as they are more cost-efficient, eco-friendly, and minimalise the use of toxic chemicals. Thus, biosynthesis is promising as a potential tool for producing NPs that are appropriate for medical purposes^3,7^. Green synthesis can be performed with the use of different organisms such as algae, plants, bacteria, and filamentous fungi. The capability to grow under various pHs, temperatures, and pressure conditions in addition to fast growth rates and easy cultivation methods make microorganisms advantageous for NP synthesis^3,8^.

Silver nanoparticles (AgNPs) are widely used in various industrial fields, including textile, cosmetics, packaging, and coatings, catalysis, or water and environmental contamination control. AgNPs possess efficient antimicrobial action against a broad spectrum of microbial species, which is regarded as the strongest efficiency among known synthesized metal NPs^7,9,10^. Hence, AgNPs are considered novel biomedicine tools for combatting infectious diseases, including ones caused by drug-resistant strains^11,12^.

Among all organisms used in NP biosynthesis, filamentous fungi seem to be most appropriate for AgNP production^6^. Fungi show high metal tolerance^12^ and produce a variety of bioactive metabolites that serve as reducing and capping agents for newly synthesized NPs. Moreover, fungal biomass is easy to cultivate under laboratory conditions and provides sufficient volume for the process in relatively short periods of time^5,8^. Mycosynthesis can be performed both intracellularly and extracellularly using extracted bioactive compounds originating from the biomass. The extracellular route is preferred for large-scale production because of the simplicity of downstream processes after synthesis^8,12,13^.

A possible use of the filamentous fungi of *Trichoderma*, *Aspergillus*, *Penicillium*, and *Fusarium* species for AgNP synthesis has been described. However, very little information is available about the use of wood decay fungi, which possess degradative capabilities^14^. White rot fungi are able to degrade lignin and plant cell wall carbohydrates using an extracellular enzyme complex. This complex is also known to be capable of degrading a variety of xenobiotics. Moreover, it has been stated that white rot fungi are capable of performing metal–NP biosynthesis. For instance, *Phanerochaete chrysosporium*, *Trametes versicolor*, and *Pleurotus sajorcaju* have been found to lead to a successful reduction of silver nitrate to silver NPs. Brown rot fungi possess an efficient lignocellulose decay system consisting of oxidases^14–16^. Information about the brown rot fungi NP synthesis is scarce. It is reported, however, that *Gloeophyllum striatum* DSM 10335 is capable of synthesizing silver NPs^14^. This topic is therefore in urgent need of further investigation.

In this paper, the antimicrobial activity and toxicity of AgNPs produced by the brown rot fungus *Gloeophyllum striatum* DSM 9592 in various process conditions have been described. Newly synthesized nanoparticles were characterized using ultraviolet/visible (UV/Vis) spectroscopy and Fourier transform infrared (FT-IR) spectroscopy, nanoparticle tracking analysis (NTA), and scanning electron microscopy (SEM). The antimicrobial activities of AgNPs were examined against a broad spectrum of bacterial strains: *Escherichia coli* ATCC 25922, *Proteus hauseri* ATCC 15442, *Pseudomonas aeruginosa* ATCC 27853, *Staphylococcus aureus* ATCC 29213, *S. aureus* ATCC 43300, *S. aureus* ATCC 6538, *S. aureus* ATCC 780699, *Campylobacter jejuni* ATCC 33560, and *Listeria monocytogenes* ATCC 19115. The toxicity evaluation procedure involved a haemolytic activity test and cytotoxicity assessment of a human fibroblast cell line.

## Results

### Biosynthesis of AgNPs using *G. striatum* and physicochemical characterization of obtained nanoparticles

**Figure 1:**
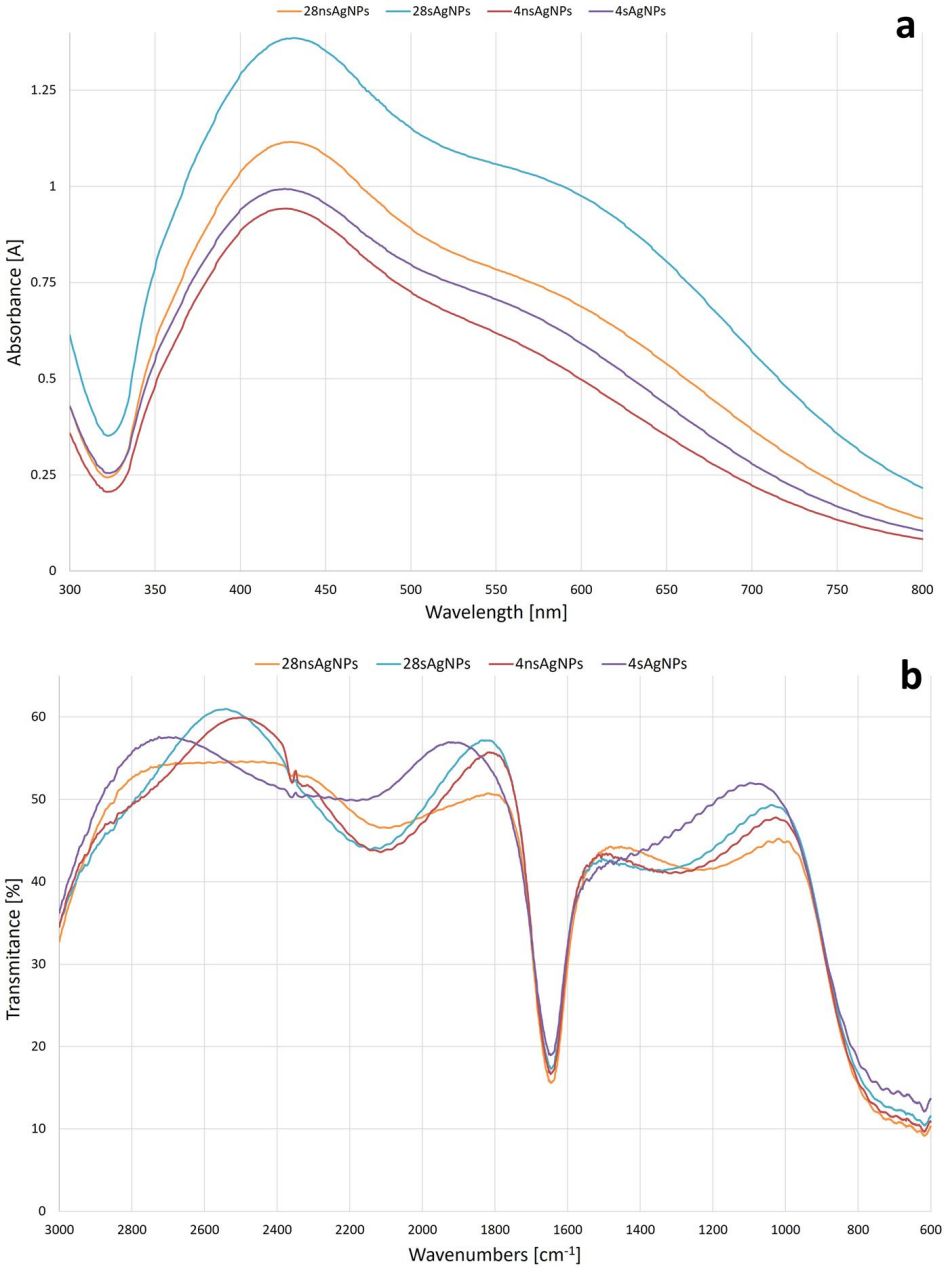
Physicochemical characteristics of the synthesized silver nanoparticles (AgNPs): (**a**) ultraviolet/visible (UV/Vis) analysis, (**b**) Fourier-transform infrared spectroscopy (FT-IR) analysis.

Biosynthesis of AgNPs was carried out extracellularly using the *G. striatum* DSM 9592 strain under four different synthesis conditions: 28 °C without shaking (28 ns AgNPs), 28 °C with shaking (28n AgNPs), 4 °C without shaking (4 ns AgNPs) and 4 °C with shaking (4 s AgNPs). The pH value of the post-culture liquid of *G. striatum* was 4.98. The successful synthesis of AgNPs under all tested conditions was proven by obtaining UV/Vis spectra of the fungal post-culture liquid supplemented with a nanoparticles precursor and checked after the process (Fig. 1a). In all cases, the maximum absorbance was detected at λ = 430 nm, which can be considered characteristic for silver NP surface plasmon resonance (SPR). The yields of AgNP production varied depending on the synthesis conditions. The highest efficiency was detected in 28 s AgNPs from which the resulting concentration of particles per milliliter was 6.46 × 10^12^. The yield of AgNP synthesis expressed as the number of particles per milliliter was then distributed as described: 2.17 × 10^12^ for 28 ns AgNPs, 3.96 × 10^12^ for 4 ns AgNPs, and 2.58 × 10^12^ for 4 s AgNPs (Table 1). FT-IR analyses showed a distinct band of 1644 cm^−1^ in all AgNP types, which differed in intensity of transmittance (Fig. 1b).

**Table 1:** The results of the nanoparticle tracking analysis (NTA).

	28 ns AgNPs	28 s AgNPs	4 ns AgNPs	4 s AgNPs
Diameter–mean [nm]	146.9 ± 2.0	160.7 ± 4.5	103.0 ± 5.7	122.1 ± 17.3
Diameter–mode [nm]	146.9 ± 2.0	121.4 ± 15.0	66.9 ± 6.9	94.0 ± 29.3
Concentration [particles/ml]	2.17 × 10^12^ ± 5.33 × 10^10^	6.46 × 10^12^ ± 4.12 × 10^11^	3.96 × 10^12^ ± 1.52 × 10^11^	2.58 × 10^12^ ± 4.58 × 10^11^
Zeta potential [mV]	− 19.42 ± 0.9399	− 20.26 ± 2.504	− 24.21 ± 1.801	− 25.19 ± 1.065
Conductivity [mS/cm]	0.1158	0.1137	0.1171	0.1139

**Figure 2:**
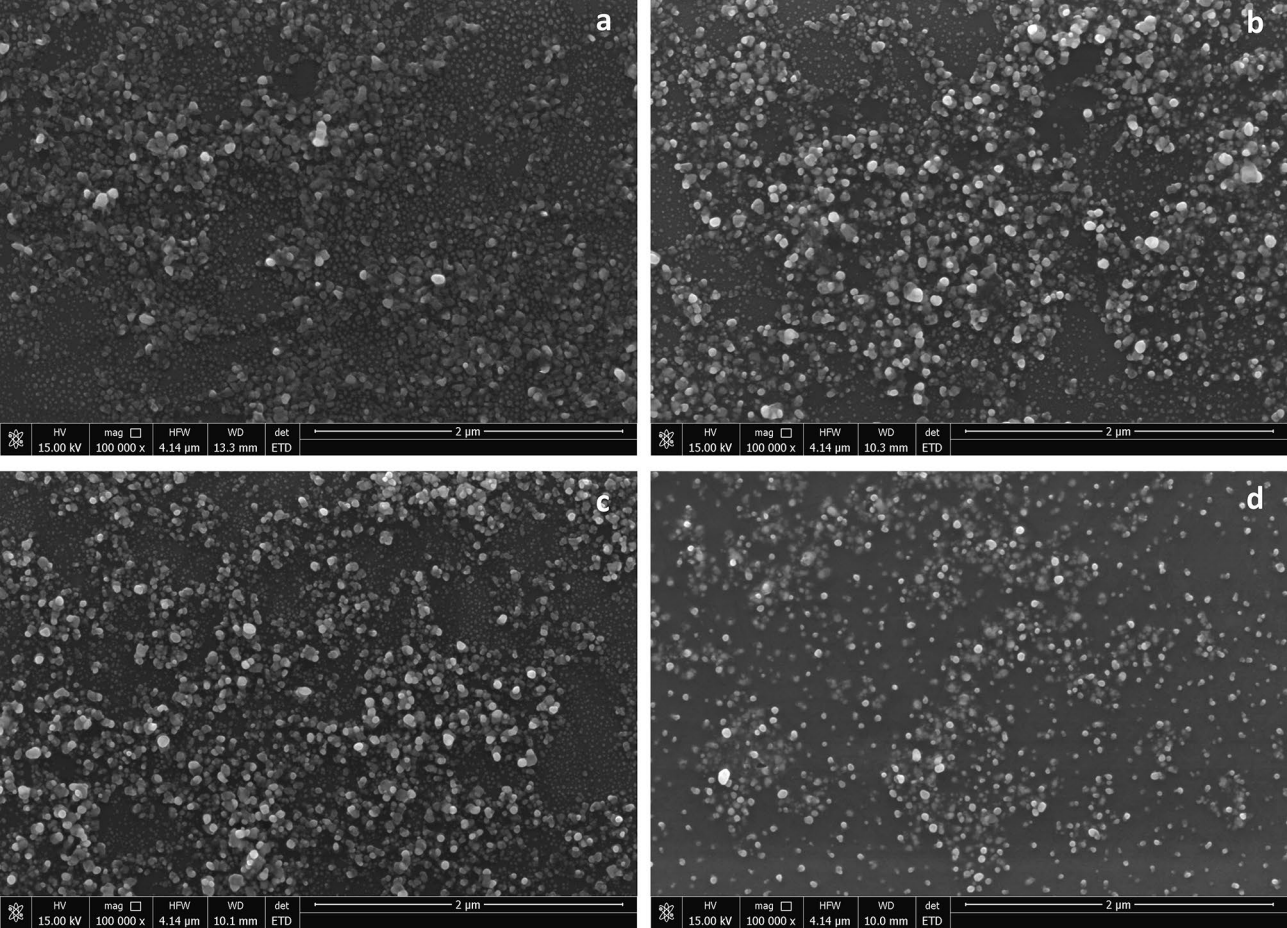
Scanning electron microscopy (SEM) images of the synthesized AgNPs: (**a**) 28 ns AgNPs, (**b**) 28 s AgNPs, (**c**) 4 ns AgNPs, and (**d**) 4 s AgNPs.

**Figure 3:**
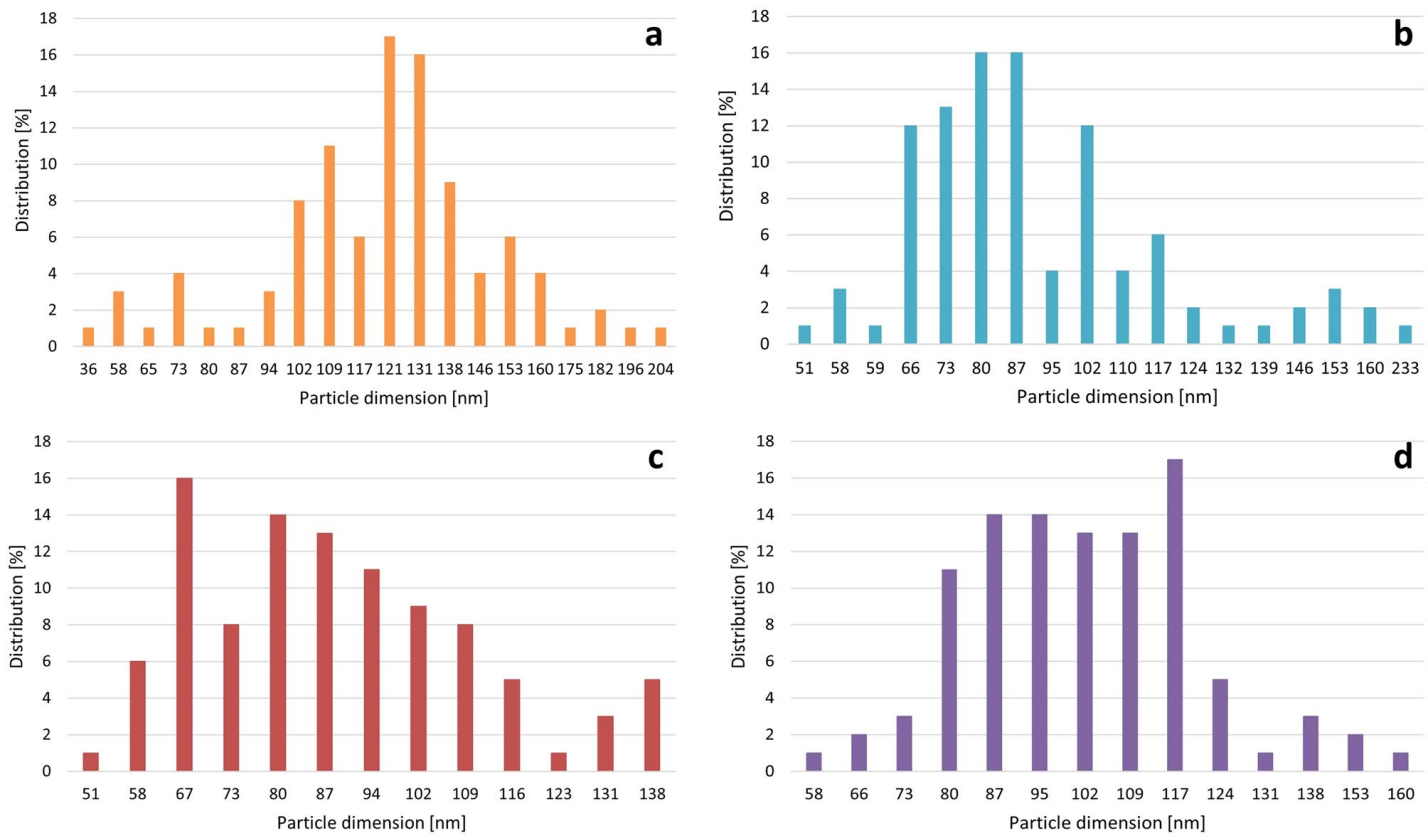
Distribution of diameter sizes of synthesized AgNPs: (**a**) 28n AgNPs, (**b**) 28 s AgNPs, (**c**) 4 ns AgNPs, and (**d**) 4 s AgNPs. Diameter sizes are given in nanometers.

SEM and nanoparticle tracking analysis (NTA) allowed estimations of the sizes of the synthesized AgNPs. These methods proved that all of the resulting AgNPs were polydispersed and varied in size among the AgNP types (Fig. 2). The diameters of the most numerous NPs of every type were 80–87, 121, 67, and 117 nm for 28 ns AgNPs, 28 s AgNPs, 4 ns AgNPs, and 4 s AgNPs, respectively, (Fig. 3).

### Determination of AgNP antibacterial activity

**Figure 4:**
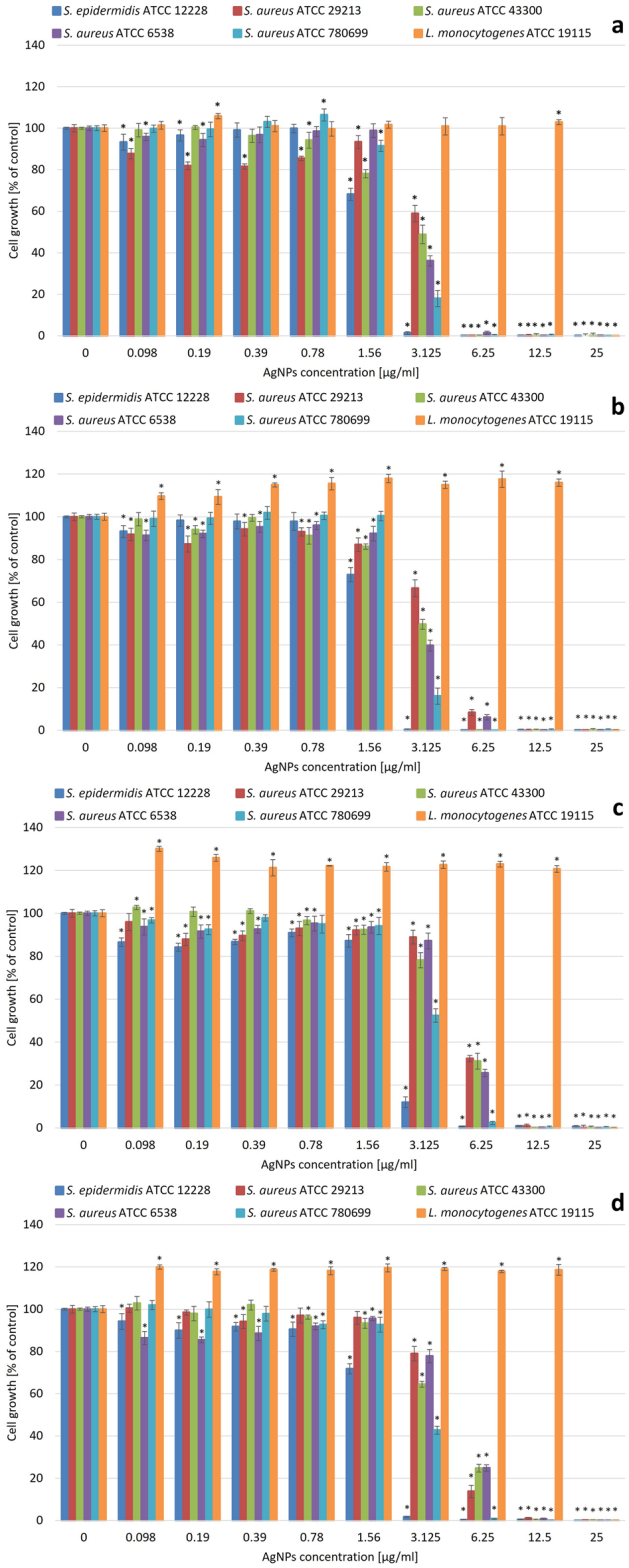
Antibacterial activity of obtained AgNPs against Gram-positive bacteria strains: (**a**) 28 ns AgNPs, (**b**) 28 s AgNPs, (**c**) 4 ns AgNPs, and (**d**) 4 s AgNPs. The results are shown as average percentage values with standard deviations of optical density (OD) of the biotic control. The statistical significance was estimated using a one-way analysis of variance (ANOVA) test with * *p* < 0.05 and is shown by an asterisk.

**Figure 5:**
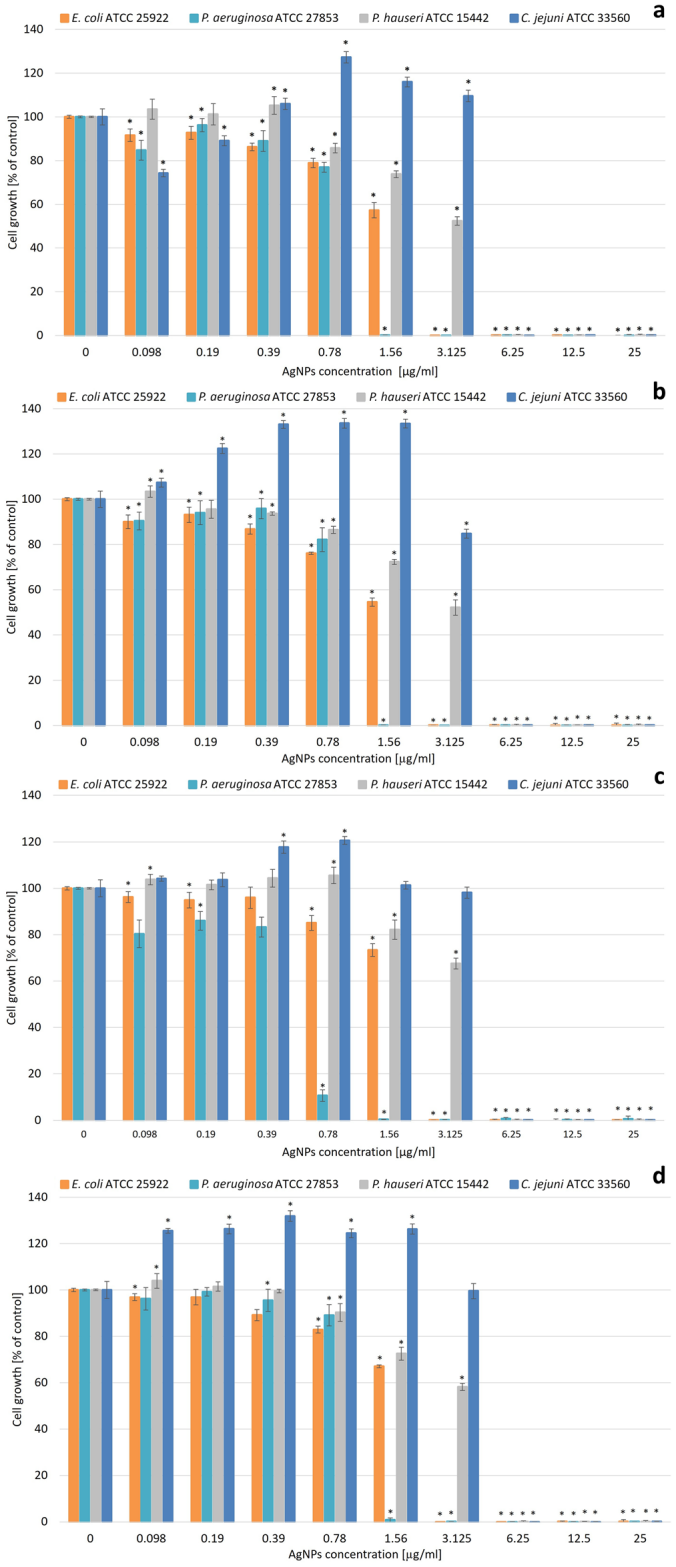
Antibacterial activity of synthesized AgNPs against Gram-negative bacteria strains: (**a**) 28 ns AgNPs, (**b**) 28 s AgNPs, (**c**) 4 ns AgNPs, and (**d**) 4 s AgNPs. The results are shown as average percentage values of optical density (OD) of the biotic control with standard deviations. The statistical significance was estimated using a one-way ANOVA test with * *p* < 0.05 and is shown as an asterisk.

The antibacterial activities of the resulting AgNPs (Figs. 4, 5) were tested in the concentration range of 0.098 to 25 µg/ml against the nine previously described bacterial strains. The results showed that Gram-negative bacterial strains were more sensitive compared to Gram-positive ones according to the obtained minimum inhibitory and minimum bactericidal concentrations (MIC and MBC, respectively) values (Table 2). The most sensitive strain was obligate aerobic *P. aeruginosa* with the MIC values reaching 1.56 µg/ml for all AgNP types although the MBC values were the highest in this case. The least susceptible Gram-negative strain was *P. hauserii* with MIC values equal to 6.25 µg/ml in all cases. Among Gram-positive bacteria strains, the most sensitive one was *S. epidermidis* with MIC values of 3.125 µg/ml for 28 ns AgNPs, 28 s AgNPs, and 4 s AgNPs and 6.25 µg/ml for 4 ns AgNPs. The MIC values associated with the *S. aureus* strains varied between 6.25 and 12.5 µg/ml depending on the NP type. The MBC values of all Gram-positive bacteria strains were comparable and exceeded the range of the tested concentration in almost all cases. The same phenomenon was detected in the strains cultivated under anaerobic conditions. The microaerophilic Gram-negative *C. jejuni* strain was more susceptible to the activity of all the tested AgNPs. MIC values were three-fold lower than those obtained for the Gram-positive facultative anaerobic *L. monocytogenes* and the MBC values were two- or three-fold lower depending on the NP type.

**Table 2:** Minimum inhibitory and minimal bactericidal concentrations (MIC and MBC, respectively, µg/ml) values of synthesized AgNPs against selected bacterial strains.

	MIC				MBC			
	28 ns	28 s	4 ns	4 s	28 ns	28 s	4 ns	4 s
*E. coli* ATCC 25922	3.125	3.125	3.125	3.125	25	3.125	6.25	3.125
*P. hauseri* ATCC 15442	6.25	6.25	6.25	6.25	6.25	6.25	> 25	12.5
*P. aeruginosa* ATCC 27853	1.56	1.56	1.56	1.56	12.5	12.5	> 25	> 25
*C. jejuni* ATCC 33560	6.25	6.25	6.25	6.25	12.5	12.5	6.25	12.5
*S. epidermidis* ATCC 12228	3.125	3.125	6.25	3.125	> 25	25	> 25	> 25
*S. aureus* ATCC 29213	6.25	12.5	12.5	12.5	> 25	> 25	> 25	> 25
*S. aureus* ATCC 43300	6.25	6.25	12.5	12.5	> 25	> 25	> 25	> 25
*S. aureus* ATCC 6538	6.25	12.5	12.5	12.5	> 25	> 25	> 25	> 25
*S. aureus* ATCC 780699	6.25	6.25	6.25	6.25	25	> 25	> 25	25
*L. monocytogenes* ATCC 19115	25	25	25	25	> 25	> 25	25	> 25

### Assessment of *P. aeruginosa* biofilm formation in the presence of AgNPs

**Figure 6:**
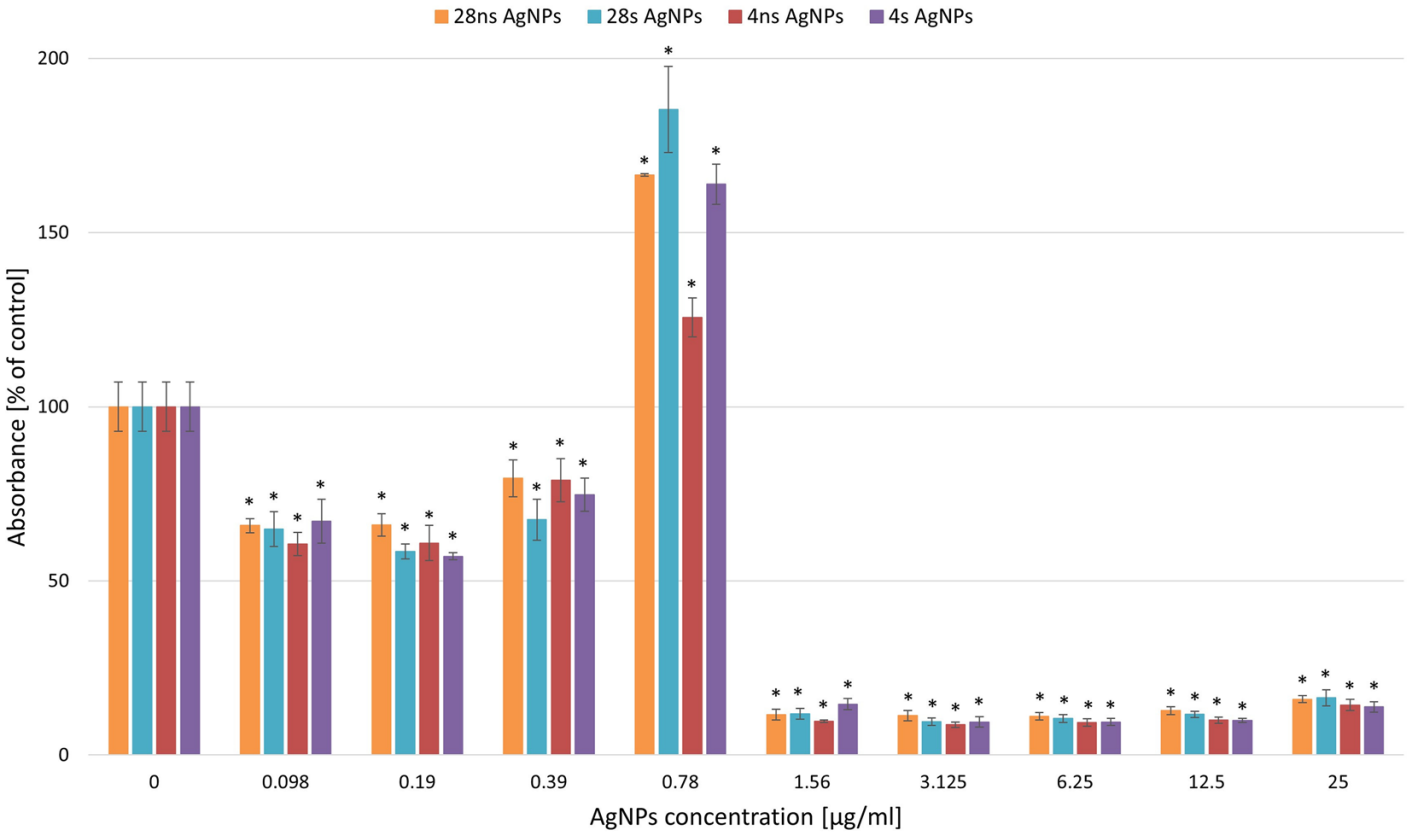
Biofilm forming ability of *Pseudomonas aeriginosa* treated with synthesized AgNPs. The results are shown as average percentage values with standard deviations of absorbance in biotic controls. The statistical significance was estimated using a one-way ANOVA test with * *p* < 0.05 and is shown as an asterisk.

The *P. aeruginosa* biofilm formation capability was tested in the presence of the resulting AgNPs in the concentration range of 0.098 to 25 µg/ml (Fig. 6). The optical density (OD) value of the biotic control was approximately 0.8648; therefore the tested strain was categorized as a moderate biofilm former^17^. It was established that biofilm formation was not affected by the presence of AgNPs in the concentrations of 0.098–0.39 µg/ml. All AgNP types caused an increase in the intensity of the biofilm formation process at a concentration of 0.78 µg/ml, reaching 150–200% of the biotic control. Higher concentrations of all AgNP types had a strong inhibitory effect on *P. aeruginosa* biofilm formation.

### Assessment of haemolytic activity of AgNPs

**Figure 7:**
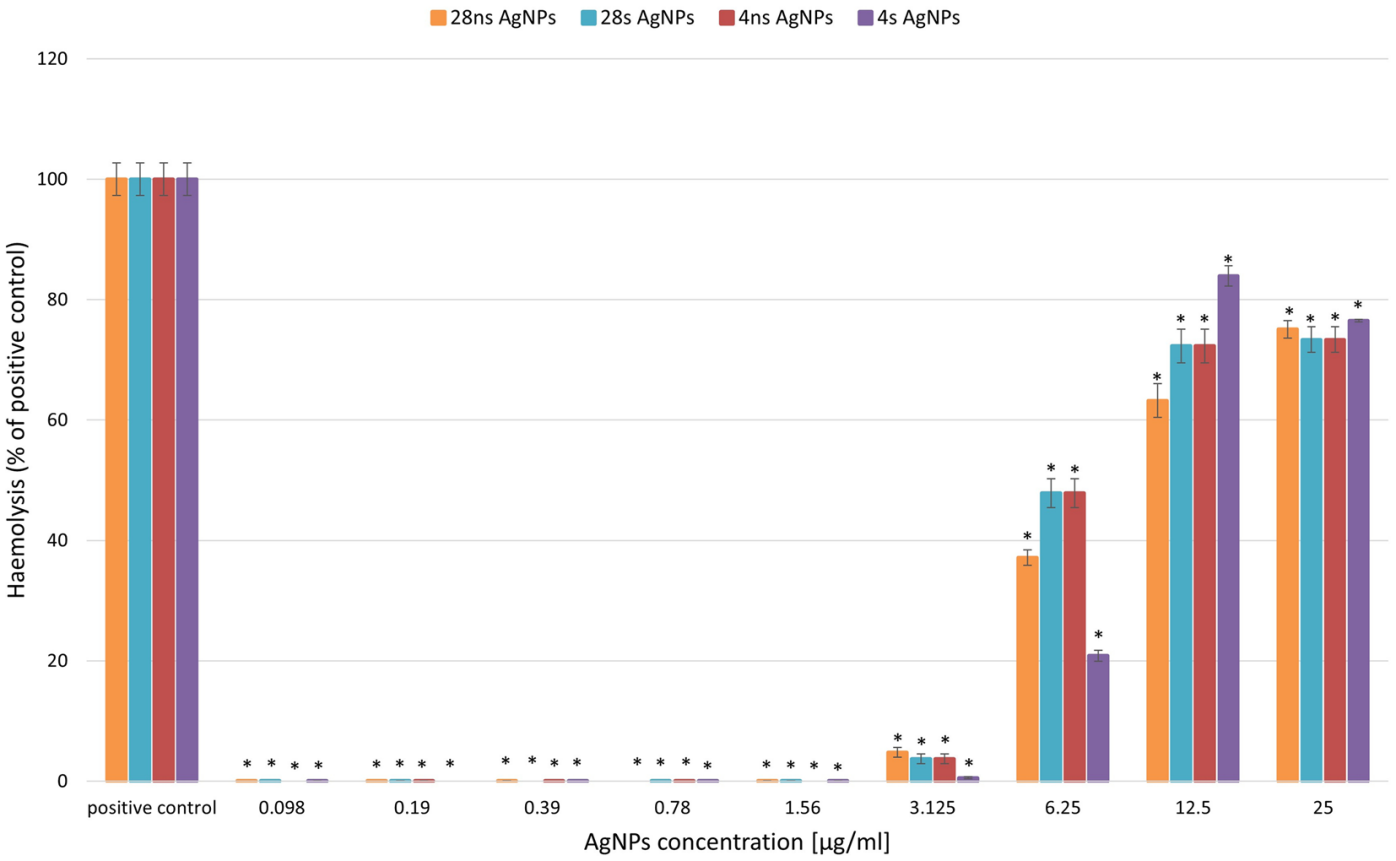
Haemolytic activity of synthesized AgNPs. The results are shown as average percentage values of absorbance with standard deviations in positive control. The statistical significance was estimated using a one-way ANOVA test with * *p* < 0.05 and is shown as an asterisk.

The haemolytic activities of all types of biosynthesized AgNPs were tested in the concentration range of 0.098–25 µg/ml, corresponding to the concentrations used in the antibacterial activity assay (Fig. 7). After 24 h of incubation, the amount of released haemoglobin was measured spectrophotometrically. The results were calculated as a percentage of positive control, which represented 100% haemolysis. None of the resulting AgNP types cause a haemolytic effect at concentrations from 0.098 to 1.56 µg/ml. A 50% haemolityc effect was detected in samples with the addition of 28 s AgNPs and 4 ns AgNPs in the concentration of 6.25 µg/ml. For higher tested concentrations, the addition of all types of the obtained silver nanoparticles caused haemolysis on the level of 60% or higher.

### Evaluation of the cytotoxic potential of AgNPs

**Figure 8:**
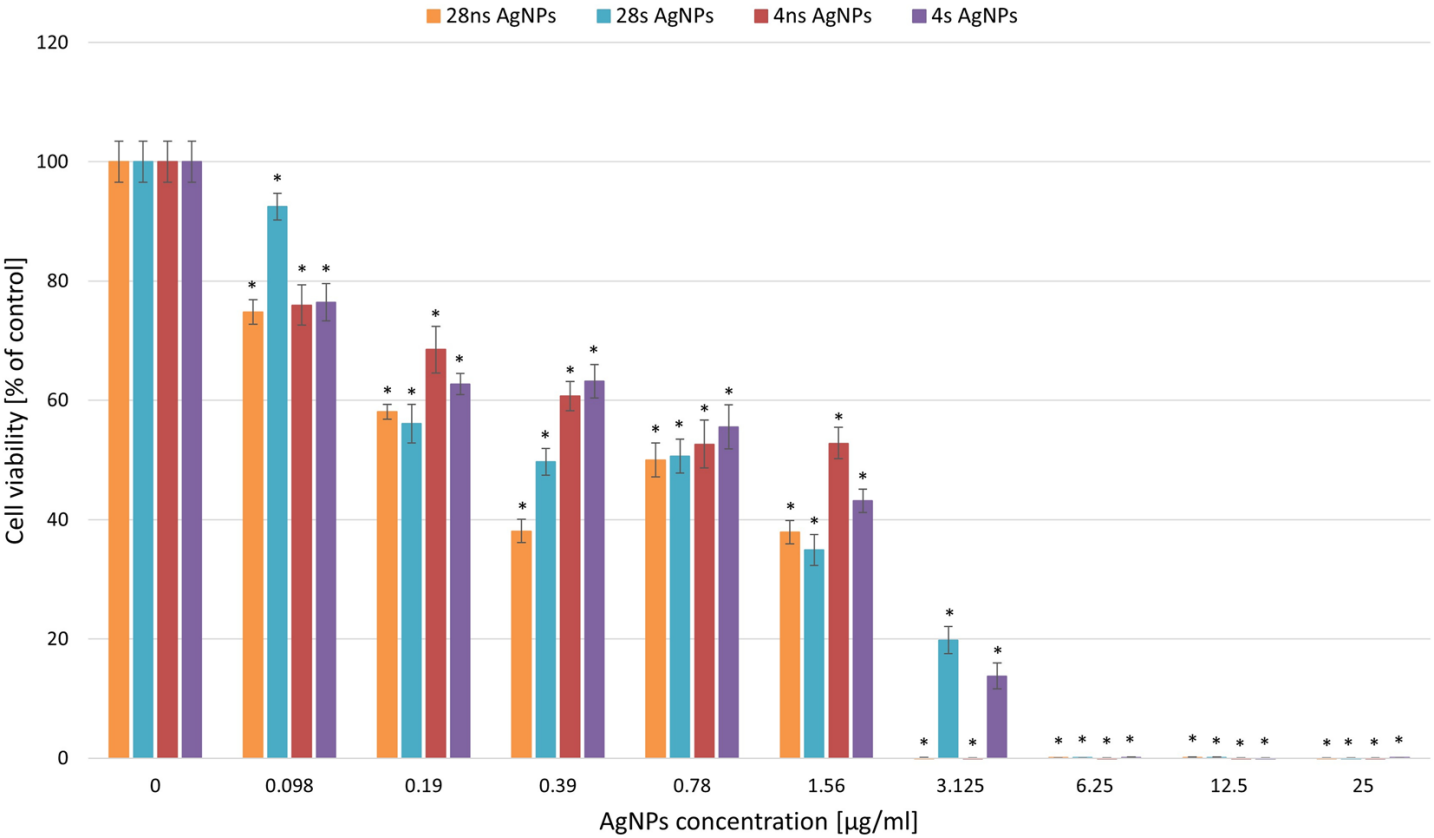
Cytotoxic activity of synthesized AgNPs. Cell viability is shown as average percentage values of absorbance with standard deviations in biotic control. The statistical significance was estimated using one-way ANOVA with * *p* < 0.05 and is shown as an asterisk.

The cytotoxic potential of all types of the resulting AgNPs was checked over the concentration range of 0.098 to 25 µg/ml, which corresponded to the concentrations used in the antibacterial activity assay (Fig. 8). The results of the cell viability assay of human fibroblasts indicated that at a concentration of 1.56 µg/ml, almost all of the tested AgNPs caused an > 50% decrease in cell viability. 4 ns AgNPs constituted an exception and were actually less cytotoxic at this concentration. At the concentration of 3.125 µg/ml, 28 ns AgNPs and 4 ns AgNPs caused a 100% decrease in cell viability. At higher concentrations, this effect was observed for all AgNP types.

## Discussion

Nanomaterials exhibit unique properties that make them a versatile tool for medicine, agriculture, and industry. Among them, AgNPs are the most thoroughly investigated. They are exploited in the production of textiles, cosmetics, sensors, and coatings, in addition to food packaging, plasmonics, and optoelectronics. They possess strong antimicrobial, anti-inflammatory, and anticancer potentials, characteristics that enable AgNPs to be used in the biomedical and pharmaceutical industries^5,7,18^. Biosynthesis of AgNPs using various organisms and filamentous fungi are of great scientific interest and relevance in terms of ‘green’ processes. They are preferable for large-scale synthesis because they can produce stable NPs. They are also easy to grow under laboratory conditions, secrete high amounts of bioactive compounds, and develop resistance to the presence of metal ions. Fungal synthesis can occur intracellularly or extracellularly; however, the latter is preferable for NP synthesis because it enhances the post-production processes^5,6,8,12,13^.

Several fungal species are known to be capable of successfully synthesizing functional AgNPs^12^. Due to their enzymatic activities, wood decay fungi are considered promising candidates NP production. White rot fungi are known to reduce silver nitrate to AgNPs^15^. However, information about AgNP synthesis using brown rot fungi is scarce. According to Zawadzka et al.^14^, the brown rot fungus *G. striatum* DSM 10335 is able to synthesize silver nanoparticles. In the present study, *G. striatum* DSM 9592, known to be capable of degrading fluoroquinolone antibiotics^19^ was selected for further investigation.

The properties of newly synthesized NPs depend on the synthesis conditions. Size distribution, shape, and surface charge seem to be crucial factors, affecting the effectiveness of NP-associated antimicrobial activity. Therefore, it is vital that the synthesis conditions are adjusted to obtain the desired product^20^. In this study, two variables affecting the process of synthesis were taken into consideration: temperature and shaking occurrence during AgNP production. It was found that both of these factors could cause differences in the resulting NPs. Maintaining high temperatures during the production process led to the synthesis of AgNPs with relatively large diameter sizes. The same tendency was observed in the case of shaking occurrence in which AgNPs synthesized under shaking conditions during the process were larger in comparison to the ones produced without shaking. The activity of synthesized AgNPs was also affected by the selected conditions. For example, AgNPs synthesized at 28 °C were more efficient against *S. aureus* strains than the ones produced at lower temperatures. In contrast, AgNPs synthesized at 4 °C without shaking were slightly less cytotoxic than the other AgNPs.

As physicochemical properties are the main factors determining the potential utility of NPs, post-synthesis characterization of the resulting AgNPs is necessary. UV/Vis analysis was chosen to confirm whether the reduction of silver nitrate in the presence of fungal post-culture liquid occurred and AgNPs were indeed synthesized. The peak of maximum absorption was found at a wavelength of 430 nm for every type of NP synthesized by *G. striatum* under different reaction conditions. Zawadzka et al.^14^ observed the peak at 425 nm in the case of silver nanoparticles synthesized by a different *G. striatum* strain. For other fungal species, the peaks in the AgNP UV/Vis spectrum were found in the range of 405–420 nm^12,13^. The peak value of 405 nm corresponded to AgNPs of plant origin^7^. SEM and NTA analyses were used to establish AgNPs size. It was found that all of the synthesized AgNPs were polydispersed. The diameter ranges of the nanoparticles in each type of AgNPs were 51–233, 36–204, 51–138, and 58–160 nm for 28 ns AgNPs, 28 s AgNPs, 4 ns AgNPs, and 4 s AgNPs, respectively. In comparison, the *G. striatum* DSM 10,335 strain had the capability of synthesizing AgNPs with an average diameter of approximately 20 nm under the synthesis conditions of 28 °C with shaking^14^. Smaller-sized AgNPs were obtained also with other fungal species, such as *Chaetomium thermophillum* and *Penicillium radiotolobatum*^12,13^ or strawberry seed extracts^7^. FT-IR spectroscopy was used to obtain insight into the chemical structure of synthesized AgNPs. It was found that every type of resulting AgNP exhibited a band of 1644 cm^-1^. This result can be considered characteristic of the C–N and C=O bonds of the amide I band. These results correspond with those obtained by Zawadzka et al.^14^ as reported in the previous study.

AgNPs have become an alternative to classic antimicrobial agents, and the bactericidal effect of AgNPs is stronger in comparison to other nanomaterials. The AgNPs develop a complex mode of action based on concurrent damage to cells at both the extracellular and intracellular levels. This phenomenon makes AgNPs effective against different groups of microorganisms, including drug-resistant strains^21–23^. Therefore, it is necessary to develop an effective method of AgNP synthesis to satisfy the need for them in various applications.

Our study showed that all types of synthesized AgNPs using the *G. striatum* DSM 9592 strain were active against the tested bacterial strains. The differences in activity levels were visible depending on the synthesis conditions; however, no specific tendency for any type of AgNP to be more effective was found. Generally, the Gram-negative strains were more susceptible with MIC values two- or three-fold lower than Gram-positive ones. The exception was the Gram-negative strain *P. hauserii* with an MIC value similar to those observed in most of the tested Gram-positive strains. In contrast, Naveen et al.^13^ found that the effectiveness of the AgNPs synthesized with *P. radiatolobatum* against anaerobic *L. monocytogenes* and *S. enterica* strains was comparable to that observed in the case of aerobic bacteria tested in our study. Moreover, their results indicated that the Gram-negative bacterium *E. coli* was less susceptible to AgNP action than Gram-positive *S. aureus*. The results from Barapatre et al.’s study on silver nanoparticles synthesized by *Emericella nidulans* confirmed that tendency. The MBC values of the resulting AgNPs in this investigation were lower for Gram-positive *S. aureus* than for the Gram-negative strains tested, which proved that the Gram-positive bacterial strains are more sensitive to AgNP activity^9^. These findings are in contrast to the results of our research. On the other hand, Hu et al.^24^ revealed that Gram-negative *E. coli* and *P. aeruginosa* were more sensitive than Gram-positive *S. aureus*, which confirms our results. Moreover, they established that *P. aeruginosa* was the most affected by silver nanoparticles synthesized with the use of the fungus from the genus *Talaromyces purpureo*.

Among all strains tested in our study, *P. aeruginosa* ATCC 27853 was the most susceptible to the activity of all types of synthesized NPs. Therefore, our research was expanded by focusing on the interactions between *P. aeruginosa* and the resulting AgNPs. *P. aeruginosa* is an opportunistic bacterium that infects patients with comorbid health problems, such as burns, cystic fibrosis, or suppressed immunity. Moreover, this specific bacterium is the cause of urinary tract infections associated with catheter implementation. A serious threat posed by *P. aeruginosa* results from its capability to form biofilms, which are defined as groups of bacteria of the same or different species that are capable of growing on several surfaces. Bacterial cells that have formed biofilms obtain properties different from those exhibited by single bacterial cells, which makes the bacteria within the biofilms very difficult to treat. Using AgNPs as surface coating agents can potentially prevent the development of *P. aeruginosa* biofilm^25,26^. In this study, the capability of *P. aeruginosa* to form biofilms was tested in the presence of our synthesized AgNPs. The results indicated that biofilm formation was strongly reduced at concentrations of 1.56 µg/ml and higher in the case of all AgNP variants. This value corresponded with the resulting MIC values. Interestingly, a strong enhancement in biofilm formation was visible in the AgNP concentration of 0.78 µg/ml even reaching up to 180% of biotic control in 28 s AgNPs. This may suggest that an increased biofilm-forming activity is a defense mechanism *P. aeruginosa* induced by the presence of AgNPs at the concentrations closest to the MIC values. LewisOscar et al.^25^ found that AgNPs synthesized with the use of *Spirulina platensis* methanolic extract also inhibited the biofilm formation by *P. aeruginosa*. In their research, the biofilm formation assay was performed in two ways: on polystyrene plates as a standard method and on the surface of latex urinary catheters (modified method). In both cases, the highest inhibition of biofilm formation was obtained with the use of the AgNP concentration of 100 µg/ml and reached 85% for the standard assay and 77% for the modified method. The effectiveness of AgNPs against *P. aeruginosa* biofilm formation has been confirmed in other studies. Shah et al.^27^ revealed that AgNPs of plant origins inhibited biofilm development at the level of 78% with the use of 8 µg/ml AgNPs. The efficacy of AgNPs against biofilm formation was confirmed also in *E. coli* and *C. albicans*^28,29^.

The possibilities for using AgNPs in different areas are increasing. Massive consumption of AgNPs in various industries has become a source of concern about the safety of AgNP exploitation^30^. It has been proven that AgNPs can cause damage to mammalian cells at several levels. Their cytotoxicity mechanisms are not fully known, but it has been reported that AgNPs can lead to an increase in reactive oxygen species production, which is believed to cause damage to DNA and other cellular compartments^10,31^. Considering the risk of AgNP-induced adverse effects on human health and the scarcity of appropriate research, further investigation into AgNP-induced cytotoxic effects is necessary.

In this study, the toxicity of synthesized AgNPs toward human red blood cells and the human fibroblast cell line was tested. The haemolytic assay revealed that all synthesized types of NPs caused at least a 60% haemolysis at the AgNP concentration of 12.5 µg/ml. At lower concentrations, haemolytic activities varied depending on the nanoparticle type. Interestingly, in the concentration of 6.25 µg/ml, 4 s AgNPs showed haemolytic activity at the level of 20%, while 28 s and 4 ns AgNPs caused almost 50% haemolysis. AgNPs obtained with the use of *G. striatum* DSM 10335 showed a lower haemolytic activity in which 66% haemolysis was induced by AgNPs at a concentration of 40 µM^14^. In the case of AgNPs of plant origin, haemolytic activity was found to be less severe. For example, AgNPs synthesized with the use of *Azadirachta indica* extract caused over 50% haemolysis at the concentration of 125 µg/ml^32^, and AgNPs obtained with *C. sativa* root extract exhibited maximal haemolytic activity at the level of 6.5% using 200 µg/ml AgNPs^33^.

The cytotoxic activity study of AgNPs synthesized by *G. striatum* revealed that three out of the four resulting NP types caused a 50% cell viability reduction at the concentration of 1.56 µg/ml in comparison to the biotic control. 4 ns AgNPs were found to be less toxic at this concentration. In the previous study, Zawadzka et al.^14^ had established that the IC_50_ value of silver nanoparticles derived from *G. striatum* DSM 10335 in murine fibroblasts was 28.76 µM, indicating that they were less cytotoxic than AgNPs used in this study. Alves et al.^12^ found that AgNPs synthesized by the fungus *C. thermophillum* were less cytotoxic in comparison to *G. striatum-*derived AgNPs. In their study, the IC_50_ value for murine fibroblast cells was obtained at an AgNP concentration of approximately 119 µg/ml, which is in contrast to the results of our research in which AgNP-associated cytotoxic effects were visible at lower concentrations. For instance, AgNPs synthesized biologically by *Bacillus* sp. showed cytotoxic effects at a concentration of 5 µg/ml thus achieving the LC_50_ value in human fibroblasts^34^.

Our research led us to draw the conclusion that modifying the conditions of the synthesis process could affect the properties of AgNPs. It was revealed that biological activity varied depending on the synthesis variant, but no specific tendency of one type of AgNP to be more bactericidal against the strains tested has been found. Gram-negative bacteria strains were generally more susceptible to the action of all tested AgNPs and *P. aeruginosa* was confirmed to be the most affected. It was also proved that the resulting AgNPs were capable of inhibiting the biofilm formation activity of this strain. Considering the high cytotoxic and haemolytic potential of the resulting AgNPs, it was confirmed that AgNPs could produce similar effects on various biological systems. The effectiveness against microorganisms is a desirable AgNP property, but the potential adverse effects of AgNPs must be considered. However, the slightly lower cytotoxic potential of 4 ns AgNPs in terms of antimicrobial activity maintained at a level comparable to that observed for the other synthesized AgNPs proves that optimisation of the synthesis process might lead to the production of effective AgNPs with reduced cytotoxicity.

## Methods

### Materials

The tested fungal strain, *Gloeophyllum striatum* DSM 9592, was obtained from the German Collection of Microorganisms and Cell Cultures GmbH (Germany). The human fibroblast BJ ATCC CRL-2522 cell line was purchased from the American Type Culture Collection (ATCC; USA). Erythrocytes used in the haemolysis assay were obtained from the Regional Center of Blood Donation and Blood Treatment in Lodz (Poland). Sabouraud dextrose broth (Difco™) and Mueller–Hinton broth (BBL™) were obtained from Becton Dickinson (Poland). Silver nitrate and 3-(4,5-dimethylthiazol-2-yl)-2,5-diphenyltetrazolium bromide (MTT) were purchased from Merck (Poland). Dimethyl sulfoxide (DMSO) and phosphate-buffered saline (PBS) were obtained from BioShop (Canada). Fetal bovine serum (FBS) and Dulbecco’s modified Eagle’s medium (DMEM) were obtained from BioWest (France). Crystal violet and acetic acid were obtained from Chempur (Poland).

### Biosynthesis of AgNPs

*G. striatum* DSM 9592 was grown on potato dextrose agar slants at 28 °C. After 18 days of cultivation, a fungal inoculum was prepared in Sabouraud Dextrose Broth supplemented with 2% glucose. The resulting *G. striatum* inoculum was incubated for 120 h at 28 °C on a rotary shaker at 120 rpm.

After incubation, fungal biomass was filtered through sterile filter paper and transferred to sterile deionized water with a volume that was equivalent to the amount of Sabouraud medium used previously. The *G. striatum* biomass was incubated for 120 h under the same conditions as previously described. Again, the mycelium was filtered through sterile filter paper to obtain post-culture fluid. The fungal filtrate was divided into four parts of equal volume. All samples were supplemented with a stock solution of silver nitrate prepared earlier on sterile deionized water. The final concentration of silver ions in each sample was 5 mM. The samples were incubated in the dark for 24 h under specific conditions: 28 °C with shaking, 28 °C without shaking, 4 °C with shaking, and 4 °C without shaking. For shaking, a magnetic stirrer was used. All samples were then stored for seven days at 4 °C.

### Characterization of synthesized AgNPs

AgNP production was checked according to the changes in the UV/Vis spectrum during silver ion reduction. Monitoring of AgNP production was conducted with the use of the UV/Vis UV/Vis spectrophotometer Specord 200 (Analytik Jena, Jena, Germany) in the absorbance mode at wavelengths from 300 to 800 nm at a resolution of 2 nm.

Biosynthesized AgNPs were examined using FT-IR spectroscopy, NTA, and SEM.

FT-IR analyses were carried out with a Nicolet™ iS50 FT-IR Spectrometer (Thermo Scientific, Waltham, MA, USA) in transmittance mode at a resolution of 0.25 cm^−1^ and spectral range of 3000–600 cm^−1^.

For NTA analyses of AgNPs, the NanoSight NS300 (Malvern Panalytical Ltd., Malvern, UK) equipped with green laser at 532 nm and NTA 3.4 Build 3.4.4 software were used. The samples were diluted 10,000 times in deionized water. In this study, zeta potential and conductivity were also determined using the Zetasizer Ultra (Malvern Panalytical Ltd., Malvern, UK).

SEM analyses were conducted with a scanning electron microscope from Quanta 250 FEG (FEI, USA). The samples were prepared by spreading AgNPs on silicon wafers, which were dried in the dark at room temperature. The images were taken in immersion mode using the Everhart–Thornley detector (ETD) at 15,00 kV acceleration at a magnification of 100,000x. The size distribution was established by measuring AgNP diameters from the SEM images with the use of the ImageJ software. The diameter was calculated based on an average of 100 NPs.

### Evaluation of antibacterial properties of synthesized silver nanoparticles

The effectiveness of synthesized AgNPs against bacteria was tested using the microdilution method according to the Clinical and Standard Laboratory Institute (CSLI) guidelines M07 (11^th^ Edition) concerning aerobic bacterial strains and M11 (9^th^ Edition) for anaerobic bacteria. The antimicrobial assay was performed with the *S. aureus* ATCC 29213, *S. aureus* ATCC 43300, *S. aureus* ATCC 6538, *S. aureus* ATCC 780699, *S. epidermidis* ATCC 12228, *E. coli* ATCC 25922, *P. hauseri* ATCC 15442, *P. aeruginosa* ATCC 27853, *C. jejuni* ATCC 33560, and *L. monocytogenes* ATCC 19115 strains. The differences in growth of all tested bacteria strains with and without AgNPs were evaluated using 96-well cell culture plates in Mueller–Hinton Broth (MHB) and Tryptic Soy Broth (TSB) for the aerobic and anaerobic bacteria, respectively. The tested AgNPs were diluted in appropriate growth medium, and the final evaluated concentrations ranged from 0.098 to 25 µg/ml in all cases. A bacterial inoculum prepared in MHB or TSB medium was added to each well to achieve a final density of 5 × 10^5^ colony-forming units (CFU)/ml and 1 × 10^6^ CFU/ml for the aerobic and anaerobic strains, respectively. Aerobic bacterial samples and adequate biotic and abiotic controls were incubated for 24 h at 37 °C. For anaerobic strains, the 96-well plates including samples, biotic and abiotic controls were placed in jars to maintain the anaerobic conditions formed using Anoxomat Mark II CTS (Mart Microbiology, B.V., the Netherlands) and then incubated for 48 h at 37 °C. After the required incubation period, the OD was measured at a wavelength of 630 nm using a Multiskan™ FC Microplate Photometer (Thermo Fisher Scientific, Pudong, Shanghai, China), and the MIC was established for each tested variant as the lowest concentration of AgNPs at which no growth of microorganisms was observed. The agar and TSB agar plates were then inoculated with 100 µl bacterial suspension taken from the wells in which no growth was observed. The agar plates with the aerobic strains were incubated for 24 h at 37 °C, and the TSB plates with the anaerobic strains were incubated under anaerobic conditions for 48 h at 37 °C to establish the MBC. The MBC was defined as the lowest concentration of AgNPs that caused a complete reduction in the viability of the tested microorganisms. Both MIC and MBC values were expressed in µg/ml.

### Assessment of the *P. aeruginosa* biofilm formation in the presence of AgNPs

The assay was carried out on 96-well plates using a scheme that was similar to one used in antimicrobial activity tests. After incubation, the media and unattached cells were removed from the wells. Each well was then washed twice with 0.85% NaCl after which 96% ethylic alcohol was added. The prepared plates were incubated for 20 min. After incubation, ethanol was removed, and the plates were left to dry. Next, a 0.1% crystal violet solution was added to all wells, and the plates were incubated for 30 min at room temperature. The stain was removed after which the wells were washed three times with 0.85% NaCl. Last, a 33% acetic acid solution was added to the dry wells, and the plates were incubated for 10 min on a rotary shaker. The absorbance was then measured at a wavelength of 600 nm using a FLUOstar Omega microplate reader.

### Evaluation of the haemolytic properties of synthesized AgNPs

The red blood cells were washed five times with phosphate-buffered saline (PBS) and then suspended in PBS in a 1:1 ratio. AgNP solutions were prepared in PBS and added to the erythrocyte samples to obtain the final AgNP concentrations ranging from 0.098 to 25 µg/ml. Moreover, negative (red blood cells in PBS without AgNPs) and positive (erythrocytes suspended in deionized water without tested NPs) controls were prepared. All samples were incubated in the dark for 24 h at 37 °C. After incubation, all samples were centrifuged for 5 min at 5000 x*g* and 4 °C. The absorbance of the resulting supernatants was measured spectrophotometrically at wavelength λ = 540 nm using a Multiskan™ FC Microplate Photometer (Thermo Fisher Scientific, Pudong, Shanghai, China). The haemolytic activities of the AgNPs was calculated based on the quantity of haemoglobin released from erythrocytes into the supernatants using the formula: $\% {\text{ Haemolysis }} = \frac{{A_{AgNPs} }}{{A_{PC} }} \times 100\%$ in which A_AgNPs_ represents the absorbance of samples incubated with AgNPs, and A_PC_ is the absorbance of positive controls, which corresponds to 100% haemolysis.

### Evaluation of the cytotoxic activity of synthesized AgNPs

The potential cytotoxic activities of the resulting fungal AgNPs were examined using the human fibroblast BJ ATCC CRL-2522 cell line. Fibroblasts at a final density of 1 × 10^4^ cells/well were cultivated in 96-well microplates in Dulbecco’s modified Eagle’s medium (DMEM) supplemented with 10% fetal bovine serum (FBS) and antibiotics (100 UI penicillin and 100 µg/ml streptomycin). The cells were incubated under conditions of 5% CO_2_ and 37 °C for 24 h. After incubation, the medium was removed, and each well was refilled with 100 µl of the fresh DMEM medium supplemented with AgNPs in the concentration range of 0.098–25 µg/ml. Corresponding biotic and abiotic controls were also prepared and incubated under the same conditions for 24 h. The medium was then removed, and 100 µl of fresh growth medium and 10 µl of 500 µg/ml MTT were added to each well. The microplates were again incubated under the same parameters for 5 h. After the final incubation, 80 µl of the solution was removed from each well after which 50 µl dimethyl sulphoxide (DMSO) was added to all wells, and the plates were left for 20 min at 28 °C to allow formazan crystals to dissolve. Spectrophotometric measurements of the cultures were obtained at λ = 595 nm using a Multiskan™ FC Microplate Photometer (Thermo Fisher Scientific, Pudong, Shanghai, China). Cell viability was calculated based on the capability to reduce MTT by the cells incubated with AgNPs compared to biotic controls.

### Statistical analysis

Each experiment was conducted in four replicates (n = 4). All results were analyzed with the use of a one-way ANOVA test with * *p* < 0.05 to estimate the statistical significance. The estimation and all needed calculations were carried out by using Excel, Microsoft^®^ Office 2021 (Microsoft Corporation, Redmont, WA, USA). The results shown in the figures are expressed as the average values with the standard deviation (SD).

## Data Availability

The data presented in this study are available on request from the corresponding author.

## References

[1] Fariq A, Khan T, Yasmin A (2017). Microbial synthesis of nanoparticles and their potential applications in biomedicine. J. Appl. Biomed..

[2] Khan I, Saeed K, Khan I (2017). Nanoparticles: Properties, applications and toxicities. Arab. J. Chem..

[3] Saravanan A (2021). A review on biosynthesis of metal nanoparticles and its environmental applications. Chemosphere.

[4] Nisar P, Ali N, Rahman L, Ali M, Shinwari ZK (2019). Antimicrobial activities of biologically synthesized metal nanoparticles: An insight into the mechanism of action. J. Biol. Inorg. Chem..

[5] Yassin MA, Elgorban AM, El-Samawaty AE-RMA, Almunqedhi BMA (2021). Biosynthesis of silver nanoparticles using *Penicillium verrucosum* and analysis of their antifungal activity. Saudi J. Biol. Sci..

[6] Cui X, Zhong Z, Runxi X, Liu X, Qin L (2022). Biosynthesis optimization of silver nanoparticles (AgNPs) using *Trichoderma longibranchiatum* and biosafety assessment with silkworm (*Bombyx mori*). Arab. J. Chem..

[7] Ali F (2022). Biosynthesis and characterization of silver nanoparticles using strawberry seed extract and evaluation of their antibacterial and antioxidant activities. J. Saudi Chem. Soc..

[8] Sudheer S (2022). Biosustainable production of nanoparticles via mycogenesis for biotechnological applications: A critical review. Environ. Res..

[9] Barapatre A, Aadil KR, Jha H (2016). Synergistic antibacterial and biofilm activity of silver nanoparticles biosynthesized by lignin-degrading fungus. BIOB.

[10] Ong WTJ, Nyam KL (2022). Evaluation of silver nanoparticles in cosmeceutical and potential biosafety complications. Saudi J. Biol. Sci..

[11] Prasher P, Singh M, Mudila H (2018). Silver nanoparticles as antimicrobial therapeutics: Current perspectives and future challenges. 3 Biotech..

[12] Alves MF (2022). Biological synthesis of low cytotoxicity silver nanoparticles (AgNPs) by the Fungus *Chaetomium thermophilum—*sustainable nanotechnology. J. Fungi.

[13] Naveen KV (2021). Fabrication of mycogenic silver nanoparticles using endophytic fungal extract and their characterization, antibacterial and cytotoxic activities. Inorg. Chem. Commun..

[14] Zawadzka K (2021). Antimicrobial activity and toxicological risk assessment of silver nanoparticles synthesised using an eco-friendly method with *Gloeophyllum striatum*. J. Hazard. Mater..

[15] He K (2017). Applications of white rot fungi in bioremediation with nanoparticles and biosynthesis of metallic nanoparticles. Appl. Microbiol. Biotechnol..

[16] Umezawa K, Niikura M, Kojima Y, Goodell B, Yoshida M (2020). Transciptome analysis of the brow rot fungus *Gloeophyllum trabeum* during lignocellulose degradation. PLoS One.

[17] Leoney A, Karthigeyan S, Asharaf AS, Felix AJW (2020). Detection and categorization of biofilm-forming *Staphylococcus aureus*, *Viridans streptococcus*, *Klebsiella pneumoniae*, and *Escherichia coli*Isolated from complete denture patients and visualization using scanning electron microscopy. Int. Soc. Prev. Community Dent..

[18] Banu AN, Kudesia N, Raut AM, Pakrudheen I, Wahengbam J (2021). Toxicity, bioaccumulation, and transformation of silver nanoparticles in aqua biota: A review. Environ. Chem. Lett..

[19] Wetzstein H-G, Schneider J, Karl W (2012). Metabolite proving fungal cleavage of the aromatic core part of a fluoroquinolone antibiotic. AMB Express.

[20] Osorio-Echevarria J, Osorio-Echevarria J, Ossa-Orozco CP, Gomez-Vanegas NA (2021). Synthesis of silver nanoparticles using white-rot fungus Anamorphous *Bjerkandera* sp. R1: Influence of silver nitrate concentration and fungus growth time. Sci. Rep..

[21] Barros CHN, Fulaz S, Stanisic D, Tasic L (2018). Biogenic nanosilver against multidrug-resistant bacteria (MDRB). Antibiotics.

[22] Burdusel A-C (2018). Biomedical applications of silver nanoparticles: an up-to-date overview. Nanomaterials.

[23] Skanda S, Bharadwaj PSJ, Darshan VMD, Sivaramakrishnan V, Vijayakumar BS (2022). Proficient mycogenic synthesis of silver nanoparticles by soil derived fungus *Aspergillus melleus* SSS-10 with cytotoxic and antibacterial potency. J. Microbiol. Methods.

[24] Hu X, Saravanakumar K, Jin T, Wang M-H (2019). Mycosynthesis, characterization, anticancer and antibacterial activity of silver nanoparticles from endophytic fungus *Talaromyces purpureogenus*. Int. J. Nanomedicine.

[25] LewisOscar F (2021). In vitro analysis of green fabricated silver nanoparticles (AgNPs) against *Pseudomonas aeruginosa* PA14 biofilm formation, their application on urinary catheter. Prog. Org. Coat..

[26] Campo-Belano C (2022). Biologically synthesized silver nanoparticles as potent antibacterial effective against multidrug-resistant *Pseudomonas aeruginosa*. Lett. Appl. Microbiol..

[27] Shah S (2019). Biofilm inhibition and anti-quorum sensing activity of photosynthesized silver nanoparticles against the nocosomial pathogen *Pseudomonas aeruginosa*. Biofouling.

[28] Miskovska A (2022). Antibiofilm activity of silver nanoparticles biosynthesized using viticultural waste. PLoS One.

[29] Selem E, Mekky AF, Hassanein W, Reda FM, Selim YA (2022). Antibacterial and antibiofilm effects of silver nanoparticles against the uropathogen *Escherichia coli* U12. Saudi J. Biol. Sci..

[30] Tortella GR (2020). Silver nanoparticles: Toxicity in model organisms as an overview of its hazard for human health and the environment. J. Hazard. Mater..

[31] de Lima R, Seabra AB, Duran N (2012). Silver nanoparticles: a brief review of cytotoxicity and genotoxicity of chemically and biogenically synthesized nanoparticles. J. Appl. Toxicol..

[32] Hawadak J, Foko LPK, Pande V, Singh V (2022). In vitro antiplasmodial activity, hemocompatibility and temporal stability of *Azadirachta indica* silver nanoparticles. Artif. Cells Nanomed. Biotechnol..

[33] Suman S (2022). Antibacterial, antioxidant, and haemolytic potential of silver nanoparticles biosynthesized using roots extract of *Cannabis sativa* plant. Artif. Cells Nanomed. Biotechnol..

[34] Khan T, Yasmin A, Townley HE (2020). An evaluation of the activity of biologically synthesized silver nanoparticles against bacteria, fungi and mammalian cell lines. Colloids Surf. B..

